# Epidemiology of Community-Acquired Respiratory Tract Infections in Patients Admitted at the Emergency Departments

**DOI:** 10.3390/tropicalmed7090233

**Published:** 2022-09-08

**Authors:** Mariana Helou, Ahmad Mahdi, Ziad Daoud, Jacques Mokhbat, Anna Farra, Elma Nassar, Ralph Nehme, Edmond Abboud, Khalil Masri, Rola Husni

**Affiliations:** 1 School of Medicine, Lebanese American University Medical Center-Rizk Hospital, Lebanese American University, Chouran, Beirut P.O. Box 13-5053, Lebanon; 2 Faculty of Medicine and Medical Sciences, University of Balamand, Tripoli, North P.O. Box 100, Lebanon; 3 Middle East Hospital, Bsalim, Mount Lebanon P.O. Box 60-387, Lebanon; 4 Centre Hospitalier du Nord, Zghorta, North P.O. Box 100, Lebanon

**Keywords:** antimicrobial resistance, respiratory infections, microbiology, epidemiology, respiratory pathogens

## Abstract

Objectives: Community-acquired respiratory infections (CARTIs) are responsible for serious morbidities worldwide. Identifying the aetiology can decrease the use of unnecessary antimicrobial therapy. In this study, we intend to determine the pathogenic agents responsible for respiratory infections in patients presenting to the emergency department of several Lebanese hospitals. Methods: A total of 100 patients presenting to the emergency departments of four Lebanese hospitals and identified as having CARTIs between September 2017 and September 2018 were recruited. Specimens of upper and lower respiratory tract samples were collected. Pathogens were detected by a multiplex polymerase chain reaction respiratory panel. Results: Of 100 specimens, 84 contained at least one pathogen. Many patients were detected with ≥2 pathogens. The total number of pathogens from these 84 patients was 163. Of these pathogens, 36 (22%) were human rhinovirus, 28 (17%) were *Streptococcus pneumoniae*, 16 (10%) were metapneumovirus, 16 (10%) were influenza A virus, and other pathogens were detected with lower percentages. As expected, the highest occurrence of pathogens was observed between December and March. Respiratory syncytial virus accounted for 2% of the cases and only correlated to paediatric patients. Conclusion: CARTI epidemiology is important and understudied in Lebanon. This study offers the first Lebanese data about CARTI pathogens. Viruses were the most common aetiologies of CARTIs. Thus, a different approach must be used for the empirical management of CARTI. Rapid testing might be useful in identifying patients who need antibiotic therapy.

## 1. Introduction

Community-acquired respiratory tract infections (CARTIs) are among the most common infections causing major morbidities and mortalities [1]. The World Health Organization recorded 1.6–2.2 million deaths caused by acute respiratory illness in children aged <5 years [2]. An accurate and immediate diagnosis of respiratory infections, which leads to timely and appropriate therapy, can improve patient prognosis [3]. Moreover, it prevents the use of unnecessary antimicrobial therapy [4,5] and decreases the spread of these infections [6]. The over-prescription of antimicrobial agents for respiratory infections is mainly caused by challenges in the clinical setting in identifying viral and/or bacterial infections [7]. The CDC reported that the number of deaths caused by multidrug-resistant infections in the United States of about 23,000 per year. However, an estimation for this was done in 2019 by Burnham et al., who estimated the number of deaths to be around 150,000 per year [8]. Antibiotics are commonly overprescribed to avoid missing a bacterial infection. In a previous study, 506 of 1000 individuals were prescribed antibiotics, and 70% of these prescriptions were inappropriate [9]. Even though most infections have viral aetiologies, 1287 antibiotic prescriptions are provided to 1000 children annually [10]. Due to the inappropriate use of antibiotics and travelling, there is a significant concern about managing the spread of emerging treatment-resistant bacteria [11]. The management of respiratory infections caused by gram-positive antibiotic-resistant bacteria (i.e., methicillin-resistant *Staphylococcus aureus*) is another challenge [12]. In addition, in the last decade, several epidemics of respiratory infections caused by novel viruses, such as coronavirus, influenza A H1N1, [11] and severe acute respiratory syndrome coronavirus 2, have emerged.

Although CARTIs have different aetiologies, they have similar clinical presentations [13]. Therefore, their diagnosis is based on appropriate laboratory testing. Conventional laboratory diagnostic methods for the routine detection of respiratory pathogens have several limitations [14]. In our country, routine cultures have low sensitivity, are time-consuming, and cannot rapidly provide a microbiological result during the early acute phase of presentation. Direct immunofluorescence assays and immune-chromatographic antigen tests can provide immediate results [14,15,16]. Molecular biology tests can be used to detect different viral and bacterial pathogens within hours [15,16]. Further, they have a high sensitivity and specificity and are, thus, reliable alternatives to other biological assays.

A literature review was performed to assess the aetiologies of CARTIs in various countries. Data were used to compare the causative agents of CARTIs between Lebanon and other countries.

Several studies worldwide identified the type of pathogens responsible for respiratory infections [17,18,19]. A study conducted at a tertiary care centre in Pakistan found that the respiratory syncytial virus (RSV) is the most common causative agent of respiratory infections in children aged <2 years during the winter season [17]. Another study analysed 1941 samples via polymerase chain reaction (PCR) for RSV infection. Results showed that 24% of the samples tested positive [18]. A surveillance study conducted in Pakistan between 2008 and 2011 showed that influenza A is the most common causative organism among people who are tested [19].

The microbiology of various causative agents is specific to each country and each region. Some similarities exist in the frequency of viral agents compared to bacterial agents. The microbiological aetiology in Asian patients with CAP differs from other regions for many reasons. This is why treatment guidelines of one country should not be used in other countries [20]. Community-acquired pneumonia (CAP) in North America is mainly caused by *Streptococcus* *pneumoniae*, *Mycoplasma* *pneumoniae*, *Chlamydia pneumoniae*, and *Legionella* [21]. In Asia, the distributions of 955 adult CAP cases according to causative agents are as follows: 29%, *S. pneumoniae*; 15%, *Haemophilus* *influenza*; 13%, *C. pneumoniae*; and 11%, *M. pneumoniae* [22]. A cross-sectional study conducted in Australia in 2019 showed that viral infections caused by viruses, such as influenza, parainfluenza, adenovirus, and RSV, account for 21% of hospitalised CAP cases [23]. However, in Asia, these viruses account for 41% of CAP cases [24]. Thus, a significant proportion of CARTIs have viral aetiologies, and clinicians still overuse antibiotics in patients with respiratory diseases. Therefore, knowing the aetiologies in our country is an important step in the One Health approach to maintaining the efficacy of available antimicrobials by decreasing inappropriate prescriptions.

Despite improvements in our knowledge of the aetiology and management of CARTIs, they remain a major cause of morbidity and mortality. Although different aetiological agents can cause these infections, a small number of agents are responsible in most cases [25]. In Lebanon, data about the type of respiratory viral and bacterial infections in the community are insufficient. Thus, this limitation can lead to the suboptimal treatment of these infections and can increase morbidity and mortality rates and the inappropriate use of antibiotics. As evident with the COVID-19 pandemic, transmission from animals and the environment has become a real threat. This is why epidemiological studies on respiratory infections are important as part of the one health approach. Hence, this study aimed to identify the causative agents of respiratory infections in patients presenting to the emergency rooms of four hospitals in Lebanon.

## 2. Methods

The current study was conducted between September 2017 and September 2018. In total, 100 respiratory specimens were collected from 100 patients presenting to the emergency departments of Center Hospitalier du Nord, Middle East Institute of Health University Hospital, Notre Dame University Hospital, and LAU Medical Center-Rizk Hospital in Lebanon. The study was approved by the Institutional Review Board of The Lebanese American University, IRB#: LAUMCRH.RH1.16/Jan/2018. All patients were diagnosed with CARTIs during the acute phase of presentation. The specimens comprised upper and lower respiratory tract samples such as nasal, nasopharyngeal, sputum, and bronchoalveolar lavage specimens. Sterile materials such as swabs and containers were used. Specimens were transported to the microbiology laboratory of the Faculty of Medicine and Medical Sciences of the University of Balamand, where the genetic material was extracted and kept at 80 °C until the multiplex PCR panel was performed for the qualitative detection of 32 respiratory pathogens.

Each specimen container was labelled with the patient’s respective code, and no personal information about the patient’s identity was included or revealed. Data on the demographic and clinical characteristics of the participants were collected and analysed using Excel sheets. A general assessment of the clinical presentation, including relevant information, such as the final clinical diagnosis, was performed.

### Molecular Experiments

All samples were extracted using NucliSENS easyMag (BioMérieux Marcy-l’Étoile, France). Each extract was subjected to multiplex PCR using the FTD^®^ Respiratory Pathogens 33 (FTD) method, which is an in vitro test with eight multiplex reverse-transcription (RT)-PCR reactions for the qualitative detection of the following viruses, bacteria, and fungi causing respiratory infections: influenza viruses A, B, and C; parainfluenza viruses 1, 2, 3, and 4; coronaviruses NL63, 229E, OC43, and HKU1; human metapneumoviruses A and B; rhinovirus; RSV A and B; adenovirus; enterovirus; parechovirus; bocavirus; cytomegalovirus; *Pneumocystis jirovecii*; *M.* *pneumoniae*; *C.* *pneumoniae*; *S.* *pneumoniae*; *Haemophilus influenzae* type B; *S.* *aureus*; *Moraxella catarrhalis*; *Bordetella* spp.; *Klebsiella pneumoniae*; *Legionella* spp.; *Salmonella* spp.; and *H.* *influenzae*. In total, 400 µL of each sample was extracted using easyMag^®^, based on the manufacturer’s instructions (BioMérieux, Marcy-l’Étoile, France). RT-PCR was run on Applied Biosystems ABI-7500 using the following conditions: 42 °C for 15 min, followed by 50 °C for 15 min, 94 °C for 3 min, and 95 °C for 10 min. Thereafter, the test was performed based on the following parameters: 40 cycles at 94 °C for 8 s and 40 cycles at 95 °C for 8 s, 60 °C for 34 s, and 60 °C for 34 s.

## 3. Results

**Figure 1:**
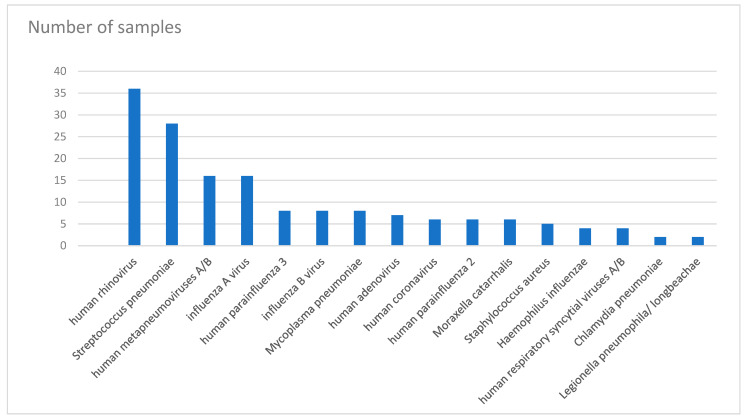
Frequency of isolated pathogens from patients recruited in this study.

In total, 100 specimens (including 69 sputum samples, 5 deep tracheal aspirates, and 24 upper respiratory tract specimens) were collected. Moreover, 84 specimens contained at least one pathogen. The numbers of samples collected from different hospitals were as follows: n = 49, Center Hospitalier du Nord; n = 26, Middle East Institute of Health University Hospital; n = 3, Notre Dame University Hospital; and n = 22, LAU Medical Center-Rizk Hospital. Of 84 positive cases, 59 involved more than two pathogens. The total number of pathogens was 163 in 84 cases. Of these, 36 (22%) involved human rhinovirus; 28 (17%), *S. pneumoniae*; 16 (10%), metapneumovirus; 16 (10%), influenza A virus; the remaining pathogens with lower percentages (Figure 1). Table 1 shows the number of detections (1, 2, 3, or 4) for each of the pathogens.

The distribution of infections was as follows: 47%, viral alone; 14%, bacterial alone; and 39%, both viral and bacterial. Viruses and bacteria were detected in 66% and 34% of all samples, respectively. These rates were comparable in the paediatric, adult, and geriatric groups.

**Figure 2:**
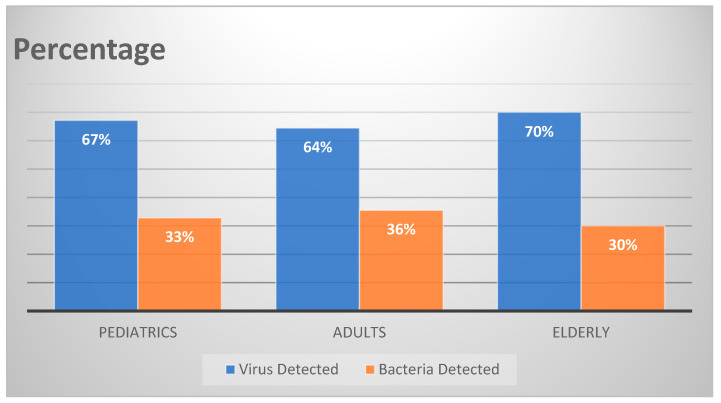
Distribution of viral and bacterial infections in different age groups.

The distribution of viral and bacterial infections was almost similar among the paediatric, adult, and geriatric groups (Figure 2).

The highest number of pathogens was observed between December and March. RSV accounted for 2% of the cases, and was only detected in paediatric patients.

**Figure 3:**
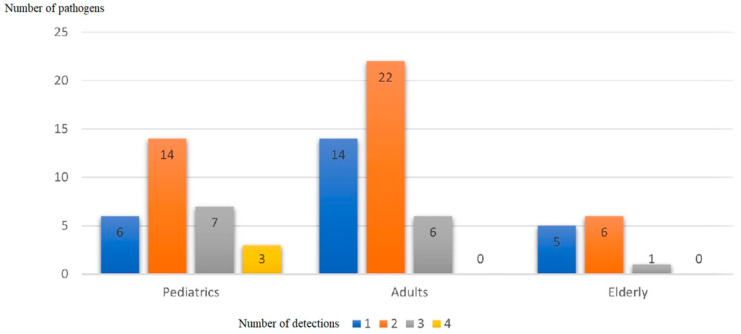
Number of pathogens detected in different age groups.

The number of any pathogen detected was almost similar between the adult, geriatric, and paediatric groups (Figure 3).

**Figure 4:**
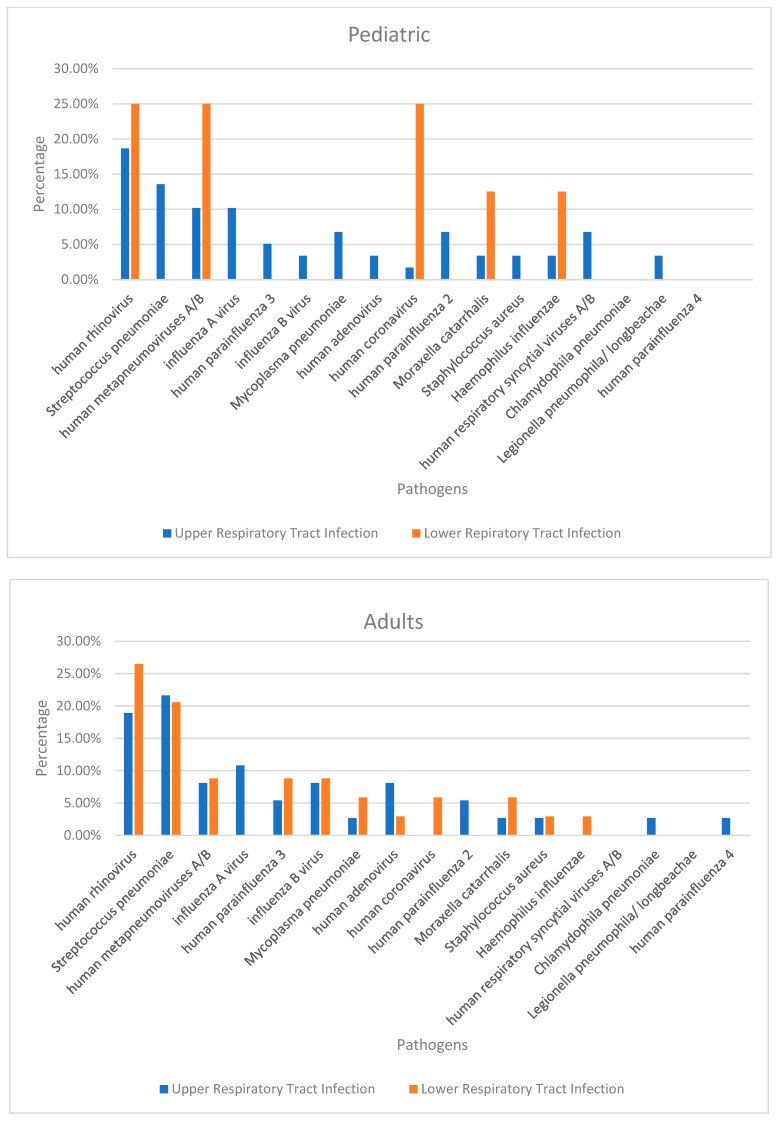

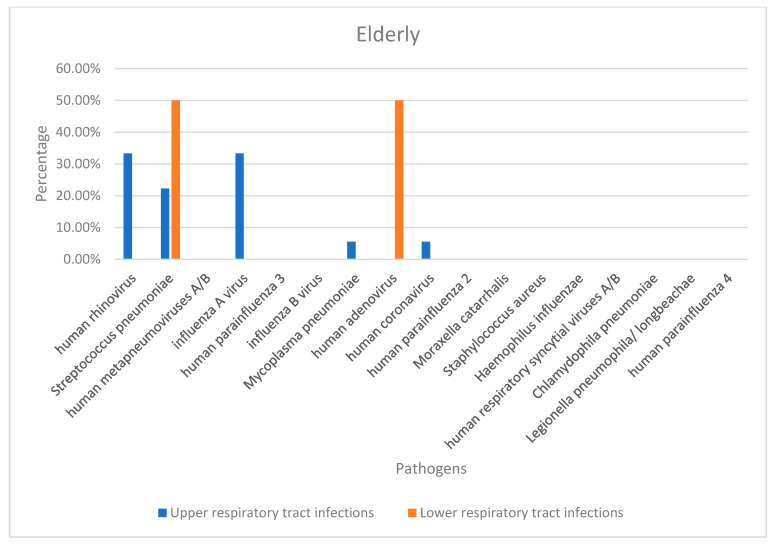
Prevalence of causative pathogens of upper respiratory tract infections and lower respiratory tract infections in paediatric, adult, and older patients.

Figure 4 shows the prevalence of the causative pathogens of community-acquired upper and lower respiratory tract infections (URTIs and LRTIs, respectively) in geriatric, adult, and paediatric patients. In the paediatric group, URTIs were caused most commonly by human rhinovirus (18.64%); LRTIs were caused mainly by human rhinovirus (25%), human metapneumoviruses A/B (25%) or human coronavirus (25%). In adults, URTIs were caused mainly by *S.* *pneumoniae* (21.62%) and LRTIs by human rhinovirus (26.47%). In the geriatric group, URTIs were caused mainly by human rhinovirus (33.33%), influenza A virus (33.33%) and LRTIs by *S.* *pneumoniae* (50%) and human adenovirus (50%).

## 4. Discussion

This study aimed to determine the causative pathogens of respiratory infections in community-dwelling patients presenting to the emergency room of four hospitals in Lebanon. CARTIs commonly had viral aetiologies. In this study, 66% of the respiratory samples contained different pathogens, including viruses. Human rhinovirus was the most commonly identified virus. It generally causes URTIs, and it can predispose patients to a superimposed LRTI. By contrast, *S.* *pneumoniae* was detected 28 times. Similar to the results of other studies, it is the most commonly identified bacterium (Figure 1).

Four different pathogens were detected in three samples. Although this finding is not common in other similar studies [26,27,28], all cases involved pediatric patients. Based on our analysis, two of three cases might have a superimposed bacterial infection caused by *S.* *pneumonia* and *S.* *aureus*. The pediatric group is more at risk of respiratory diseases than the adult and geriatric groups. The infections might have been caused by primary and secondary pathogens, which explains the higher frequency of pathogen detection. These pathogens could be associated with a previous respiratory infection or possible colonisation in cases of bacterial infections.

Globally, pneumonia is still associated with high mortality and morbidity rates and healthcare-related costs [1,2,8]. Despite advancements in CAP management, the identification and treatment of causative agents remain a challenge. Moreover, although the incidence of CAP decreased after the introduction of polysaccharide vaccines, pneumococcus is still the most frequent causative pathogen of pneumonia in the USA [26]. Moreover, intracellular pathogens such as *M.* *pneumoniae*, *C.* *pneumonia*, and *L.* *pneumoniae* commonly cause CAP. Contrastingly, viral aetiologies account for 7–36% of CAP cases [26]. A meta-analysis showed that 22% of adult CAP cases in Europe have viral aetiologies [27].

Based on a previous study, viruses are the most common cause of URTIs and LRTIs, followed by atypical bacteria and bacterial pathogens [28]. This finding is in accordance with ours. Via further investigation of viral pathogens, a systemic review examined the etiologic pathogens causing acute respiratory infections in older adults. Results showed that parainfluenza viruses, human metapneumoviruses, RSV, influenza viruses, adenoviruses, rhinoviruses, and coronaviruses are important causes of acute respiratory infections [29]. Another study reported that the prevalence of viral and combined viral–bacterial CAP is high in hospitalised school-age children. This result supports the notion that the presence of a virus, acting either as a direct or an indirect pathogen, may be the rule rather than the exception in CAP development in school-age children requiring hospitalisation [30]. Our study had similar findings. That is, viral infections were commonly observed in the paediatric, adult, and geriatric groups. In addition, coinfection is still the most common type of infection in these groups.

The most common causative agents of URTIs and LRTIs in paediatric, adult, and geriatric patients between Lebanon and other countries were compared (Figure 4). In France, older patients commonly experience URTIs caused by human rhinoviruses and influenza A virus. These results are similar to ours. However, *S. pneumoniae* was a common causative pathogen [31]. Moreover, it was a major causative bacterium of URTIs in adult patients in the USA and Jordan, as in Lebanon [32,33]. Meanwhile, rhinovirus was the main causative pathogen in the USA, as in Lebanon, for the specified category [32]. Rhinoviruses are the most common viral aetiologies of URTIs in paediatric patients in Lebanon. In contrast, the main causes of bacterial LRTIs in adults were *S. pneumoniae* in the USA, Europe, and Lebanon, *H. influenzae* in Turkey, *M. pneumoniae* in Canada, and *M. pneumoniae* in Malawi [31,32,34,35,36,37]. Meanwhile, influenza viruses were the main cause of LRTIs for this category in Malawi. However, it was human rhinovirus in our study [34]. *C. pneumoniae* was the main causative bacterium of URTIs in paediatric patients in Jordan. Meanwhile, it was *S. pneumoniae* in our study despite the low adult vaccination rates in both countries [33]. Another research showed that compared with other countries, Canada has a lower prevalence of *S. pneumoniae* causing LRTI in adults (Table 2) [36]. This finding might be attributed to the high vaccination rates against *S. pneumoniae*. That is, 88% of adults are up to date on their vaccination schedule [38].

Our study showed the common causative agents of URTIs and LRTIs in paediatric, adult, and older populations. The results were comparable to those of studies conducted in nearby and other countries [31,32,33,34,35,36,37]. Hence, they could be used as a guide for the empiric treatment of RTIs in Lebanon and its neighbouring countries. New studies are being conducted in some specific regions, and the pathogens detected might be different from the previous studies mentioned. A study conducted in rural areas of the Philippines showed that *Haemophilus influenzae* (12%) was the most commonly detected bacteria and influenza virus (5%) the most commonly detected virus [39]. Another study in adults having a CAP in Zambia found that *Mycobacterium tuberculosis* was the most common isolate (20%), followed by *Candida* species (18%), *Klebsiella pneumoniae* (12%), and *Pseudomonas aeruginosa* (7%) [40].

The current study had some limitations. That is, epidemiological data were not evaluated. Accordingly, the association between the characteristics of patients and disease course and aetiology was not examined. Finally, radiological findings were not assessed.

## 5. Conclusions

This study initially provided data about the causative pathogens of CARTIs among Lebanese. Viruses were the most common aetiologies of CARTIs in the paediatric, adult, and geriatric groups. Coinfection was the most frequent type of infection, and *S. pneumoniae* was also commonly detected. Thus, a different approach must be used for the empirical management of CARTI. It should be based on rapid testing, which can identify the patient group who will benefit from antibiotic therapy. It is a useful tool for antimicrobial stewardship. It can help improve and limit the misuse of antibiotics in this patient group, which is part of the One Health approach that aims to mitigate antimicrobial resistance. Further epidemiological studies during and after the COVID-19 pandemic are needed to better understand the dynamics of transmission between humans, animals, and the environment.

## Figures and Tables

**Table 1:** Number of detections for each pathogen.

Pathogen	Total Number	Number of Detections			
		1 detection	2 detections	3 detections	4 detections
human rhinovirus	36	2	23	8	3
*Streptococcus pneumoniae*	28	6	16	4	2
human metapneumoviruses A/B	16	5	5	5	1
influenza A virus	16	6	7	3	0
human parainfluenza 3	8	1	2	4	1
influenza B virus	8	3	4	1	0
*Mycoplasma pneumoniae*	8	2	3	3	0
human adenovirus	7	0	4	3	0
human coronavirus	6	0	3	2	1
human parainfluenza 2	6	0	1	4	1
*Moraxella catarrhalis*	6	0	3	2	1
*Staphylococcus aureus*	5	0	3	1	1
*Haemophilus influenzae*	4	0	2	2	0
human respiratory syncytial viruses A/B	4	0	2	1	1
*Chlamydia pneumoniae*	2	0	1	1	0
*Legionella pneumophila*/longbeachae	2	0	2	0	0
human parainfluenza 4	1	0	1	0	0

**Table 2:** Prevalence of causative pathogens of upper and lower respiratory tract infections in paediatric, adult, and older patients in the USA, Canada, Europe, France, Malawi, Turkey, and Jordan.

Aetiology of Respiratory Tract Infections								
Source	Country	Sampling Technique	Upper Respiratory Tract Infections				Lower Respiratory Tract Infections	
			Elderly	ADULTS		Pediatric	Adults	
			Viral Aetiology	Bacterial Aetiology	Viral Artiology	Bacterial Aetiology	Bacterial Aetiology	Viral Aetiology
[32]	USA	not mentioned		Presumed virus or chlamydia 30–40% (not tested)			*Streptooccus pneumonia*	
				Group A Streptococci 5–10%	rhinovirus 25–30%		Enteric gram-negative organisms	
				Mycoplasma 5–10%	coronavirus > 10%		*Staphylococcus aureus*	
					Influenza Virus, RSV, Adenovirus and Parainfluenza Virus 10–15%		*Hemophilus influenza*	
					Other viruses		*Pseudomonas aeruginosa*	
[36]	Canada	Blood culture					Unknown 51.6%	
		sputum culture					*Mycoplasma pneumoniae* 15%	
		acute and convalescent serum samples for serology					*Chlamydia pneumoniae* 12%	
		Antibodies to *Mycoplasma pneumoniae* and *Chlamydia pneumoniae* determined using enzyme-linked immunosorbent assays					*Streptococcus pneumoniae* 5.9%	
							*Haemophilus influenzae* 4.9%	
							*Chlamydia pneumoniae* and *Mycoplasma pneumoniae* 2.1%	
							*Haemophilus parainfluenzae* 1.9%	
							*Staphylococcus aureus* 1.1%	
							*Moraxella catarrhalis* 1.1%	
							*Streptococcus* species 0.9%	
							Other 2.8%	
[37]	Europe (UK, Spain and Sweden)	not mentioned					No pathogen identified 49.8%	viruses 11.7%
							*Streptococcus pneumoniae* 19.3%	
							*Mycoplasma pneumoniae* 11.1%	
							*Chlamydia pneumoniae* 8%	
							*Haemophilus influenzae* 3.3%	
[31]	France	QiaAmp MinElute virus spin kits	Influenza A (H3N2)					
		real-time Reverse Transcription quantitative PCR (RT-qPCR)	Human rhinovirus 16%					
			Human coronavirus OC43 7%					
			Respiratory Syncytial Virus 5%					
			Human metapneumovirus 5%					
			Influenza B/Victoria 5%					
[34]	Malawi	blood culture					No pathogen detected 39.4%	Influenza viruses 8.8%
		*Streptococcus pneumoniae* urinary antigen detection					*Mycobacterium tuberculosis* 23%	Adenovirus 7.7%
		sputum mycobacterial culture					*Streptococcus pneumoniae* 21.4%	Coronaviruses 6.8%
		Xpert MTB/RIF					Nontuberculous mycobacteria 2.9%	Parainfluenza viruses 3.7%
		nasopharyngeal aspirate multiplex PCR					*Salmonella enterica* serovar Typhi 2.2%	Rhinovirus 4.2%
							Nontyphoidal *Salmonella* 1.6%	Bocavirus 2.9%
							*Mycoplasma pneumoniae* 1.3%	Metapneumovirus 2.0%
							Other gram-negative enteric bacilli 0.7%	RSV 1.8%
							*Staphylococcus aureus* 0.4%	Enterovirus 1.1%
							*Chlamydia pneumoniae* 0.4%	Parechovirus 1.1%
[35]	Turkey	sputum cultures					*Haemophilus influenzae* 44.9%	
							*Streptococcus pneumoniae* 25.5%	
							*Moraxella catarrhalis* 12.2%	
							*Pseudomonas aeruginosa* 3.1%	
							*Klebsiella* spp. 1%	
							*Haemophilus parainfluenzae* 1%	
							*Staphylococcus aureus* 1%	
[33]	Jordan	sputum cultures		*Streptococcus pneumoniae* 26%		*Chlamydia pneumoniae* 14%		
				*Chlamydia pneumoniae* 23%		*Mycoplasma pneumoniae* 6%		
				*Haemophilus influenzae* 17%		*Streptococcus pneumoniae* 3%		
				*Mycoplasma pneumoniae* 9%		*Haemophilus influenzae* 3%		
				*Legionella pneumophila* 6%		*Pseudomonas aeruginosa* 3%		
				*Klebsiella pneumoniae* 6%				

## Data Availability

Data is available from authors.
